# Therapeutic effects of virtual reality video gaming on functional mobility, balance, and gait speed in individuals with tropical spastic paraparesis: A randomized crossover clinical trial

**DOI:** 10.1590/0037-8682-0623-2020

**Published:** 2021-01-29

**Authors:** Erika Pedreira da Fonseca, Katia Nunes Sá, Rebeca Freitas Reis Nunes, Camille Rosa de Jesus Souza, Mayra Castro de Matos Sousa, Elen Beatriz Pinto

**Affiliations:** 1 Universidade Católica do Salvador, Departamento de Fisioterapia, Salvador, BA, Brasil.; 2 Escola Bahiana de Medicina e Saúde Pública, Departamento de Pós-Graduação, Salvador, BA, Brasil.

**Keywords:** Gait, Tropical spastic paraparesis, Mobility limitation, Postural balance, Virtual reality

## Abstract

**INTRODUCTION:**

Individuals with human T-cell lymphotropic virus 1-associated myelopathy/tropical spastic paraparesis (HAM/TSP) experience sensorimotor alterations, which can affect functional performance. Virtual reality (VR) videogaming is a therapeutic option, though there is scarce evidence for its use in this population. We aimed to investigate the therapeutic effects of a VR video game on functional mobility, balance, and gait speed in individuals with HAM/TSP.

**METHODS:**

We conducted a blinded, crossover clinical trial comprising 29 individuals with HAM/TSP and randomized them into two groups: (1) early therapy: rehabilitative protocol started immediately after the initial evaluation and (2) late therapy: rehabilitative protocol started 10 weeks later. We assessed all participants for balance using the Berg Balance Scale (BBS) scores, functional mobility using the Timed Up and Go (TUG) test, and gait speed using video camera and CvMob software. Differences were considered significant if *p*<0.05.

**RESULTS:**

The early therapy group individuals presented with higher BBS scores (*p*=0.415), less TUG times (*p*=0.290), and greater gait speed (*p*=0.296) than the late therapy group individuals.

**CONCLUSIONS:**

VR videogaming is a useful option for rehabilitative therapy in individuals with HAM/TSP; it positively affects balance, functional mobility, and gait speed.

## INTRODUCTION

Human T-cell lymphotropic virus 1 (HTLV-1)-associated myelopathy/tropical spastic paraparesis (HAM/TSP) is a neurological disorder characterized by demyelination of the central nervous system, predominantly the spinal cord^1^ ^-^ ^5^. Initial signs of HAM/TSP include a reduction in muscular strength, lower-limb spasticity, and sphincter disorders^6^. This disease results in functional motor, sensory, and autonomic dysfunctions that lead to changes in gait, impaired balance, and loss of functional mobility, thereby increasing the risk of falling^1^ ^-^ ^5^. Additionally, these physiological alterations may be influenced by fatigue arising from depressive symptoms as well as diminished social interactions, sense of well-being, and physical fitness^7^ ^,^ ^8^.

HTLV-1 infects an estimated 20 million people worldwide. In Brazil, this infection is endemic with the highest incidence reported in Salvador^9^, the capital of the northeastern state of Bahia. Secondary to HTLV-1 infection^10^, 3-5% of affected individuals develop HAM/TSP, with a higher prevalence in women aged ≥ 40 years^6^ ^,^ ^9^. Nevertheless, HTLV-1 remains a neglected disease, and rehabilitation in HAM/TSP is crucial to promote functional improvement in affected individuals^4^ ^,^ ^11^ ^,^ ^12^.

Among the different rehabilitative strategies, studies have demonstrated virtual reality (VR) videogaming as a promising therapeutic option for motor and cognitive rehabilitation in patients with neurological impairments^13^ ^,^ ^14^, including demyelinating diseases such as HAM/TSP^4^ ^,^ ^15^ ^,^ ^16^ and multiple sclerosis^16^. However, evidence regarding the impact of VR in HAM/TSP rehabilitation remains unclear. The present study aimed to investigate the effects of rehabilitation via VR videogaming on functional mobility, balance, and gait speed in HAM/TSP patients, considering the importance of these factors in fall prevention.

## METHODS

The present randomized crossover clinical trial (ClinicalTrials.gov: NCT02877030) involved patients aged 18-64 years with independent gait, whose conditions were diagnosed as HAM/TSP according to the criteria of the World Health Organization^17^. We excluded patients who were pregnant; presented with other neurological conditions, psychiatric disorders, rheumatic or orthopedic diseases; had previously undergone lower-limb amputation; or had difficulty in understanding the instruments used for the evaluation. This trial was part of a larger study approved by the local institutional review board (CAAE 49634815.2.0000.5628). All patients provided written informed consent.

In accordance with the CONSORT (http://www.consort-statement.org/) guidelines, a third party randomized the patients into two groups, late treatment (LT) group and early treatment (ET) group, using the online Random software (https://www.random.org/). Blinded and previously trained investigators evaluated the patients 10 and 20 weeks after the initial assessment, with a 1-week washout period after group crossover^18^. For analysis, data from the LT group at the second assessment time point were used as controls for ET group, while ET data were used as controls for LT following the final assessment. Patients were asked to maintain their usual activities, including rehabilitation, throughout the evaluation time points.

Patients were evaluated for balance and functional mobility using the Berg Balance Scale (BBS) scores^19^ and the Timed Up and Go test (TUG)^20^ ^-^ ^22^, respectively. Habitual gait velocity was filmed using a GoPro HERO 3.0^®^ camera and analyzed with CvMob software^23^ ^,^ ^24^. In addition, patients answered a demographic questionnaire and provided a history of falls in the last 3 months, defined as “inadvertent fall to the ground or a lower level, excluding intentional changes in position using furniture, walls, or other objects as support”^25^. 

**FIGURE 1:**
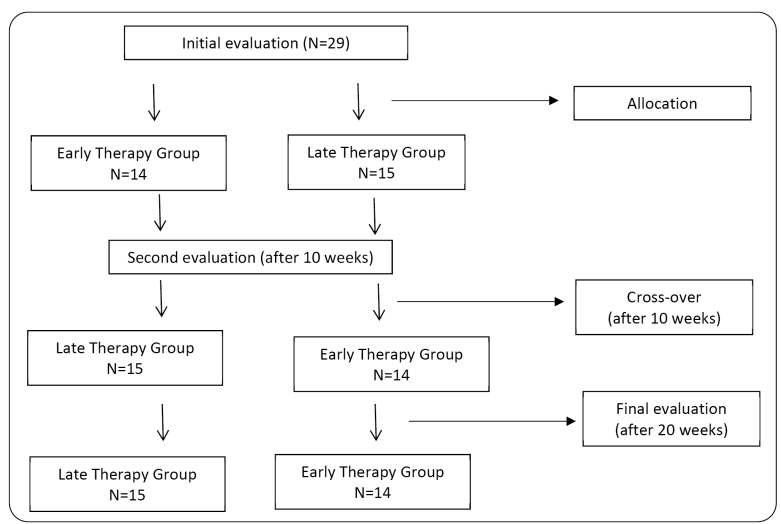
Flowchart of the study design in accordance with the CONSORT guidelines.

The ET group began the sensorimotor exercise protocol immediately after the initial assessment, whereas the LT group, considered as a control group for ET during this initial phase, initiated the protocol only at the beginning of the second 10-week study period. After this time point, the ET group received no therapeutic intervention, constituting the control phase. The ET and LT groups performed sensorimotor exercise sessions lasting 20 min, twice a week for 10 consecutive weeks: ET from weeks 1 to 10, and LT from weeks 10 to 20, following the 1-week washout period (Figure 1). We connected a VR videogame to a Nintendo Wii® console, in which arrows were randomly cast toward the player from above. Players were asked to move in the direction of the arrows to induce movements that displaced their body’s center of pressure using the Nintendo Wii® platform. Initially, arrows appeared at 3-s intervals, with a progressive increase in the appearance of arrows over the rehabilitation treatment period, demanding greater weight-shift from the participants. Patients who reported fatigue during a session were allowed to rest; resting periods were not deducted from the overall duration of 20 min per session.

Statistical analysis was performed using SPSS Statistics for Windows, version 17 (SPSS Inc., Chicago, IL, USA). The demographic and functional characteristics of participants were described with qualitative variables expressed using absolute and relative frequencies. The Shapiro-Wilk test was used to determine the distribution of these variables. The normally distributed variables were described as means and standard deviations, while skewed variables were described as medians and quartiles. We used Student’s t and Fisher’s exact tests to compare the demographic and functional characteristics. The treatment effect between groups and confidence intervals were assessed using Cohen’s d, with values compared with the reference data previously published by Rosenthal et al. (1996)^26^. To evaluate differences in functional mobility, balance, and gait speed between the control and therapy phases of ET and LT groups, repeated-measures ANOVA testing was applied. A *p* < 0.05 was considered significant. 

The necessary sample size of 12 participants in each group was estimated using the online tool provided by the Laboratory of Epidemiology and Statistics of the University of São Paulo (LEE-USP; http://www.lee.dante.br/), considering a study power of 80% and 5% alpha level, standard deviation (SD) of 3, and a 3-s difference detection limit between TUG values^4^. Moreover, 20% more participants were included in each group, considering possible losses.

## RESULTS

We enrolled 29 patients with HAM/TSP, with therapeutic intervention and evaluations performed between April and December 2017 (Figure 1). Table 1 lists the demographic and functional characteristics of these patients. The analysis revealed no significant differences between ET and LT groups.

**TABLE 1:** Demographic and functional characteristics of patients (N=29) with HAM/TSP.

Variables	Total	Early therapy	Late therapy	***p* value**
	(N=29)	(n=14)	(n=15)	
Age in years (mean ± SD)^a^	51.02±9.83	46.89±11.43	52.27±7.95	0.23
Sex: Female n (%)^b^	16 (55%)	9 (66.7%)	7 (45.5%)	0.40
Use of walking aid, n (%)^b^	15 (50%)	8 (55.6%)	7(45.5%)	1.00
History of falls, n (%)^b^				
None	4 (15%)	3 (22.2%)	1 (9.1%)	0.38
One	4 (15%)	0 (0.0%)	4 (27.3%)	0.38
Two	2 (5%)	1 (11.1%)	0 (0.0%)	0.38
Three or more	19 (65.0%)	10 (66.7%)	10 (63.6%)	0.38
Berg Balance Scale score (mean ± SD)^a^	45.24±9.96	41.56±7.93	44.18±6.41	0.42
Timed Up and Go - seconds (mean ± SD)^a^	17.44±11.29	22.19±12.72	18.13±6.87	0.37
Gait speed - meters/seconds (mean ± SD)^a^	0.21±0.9	0.22±0.09	0.22±0.09	0.89

SD: standard deviation. ^a^ t-test; ^b^ Fisher’s exact test.

**FIGURE 2:**
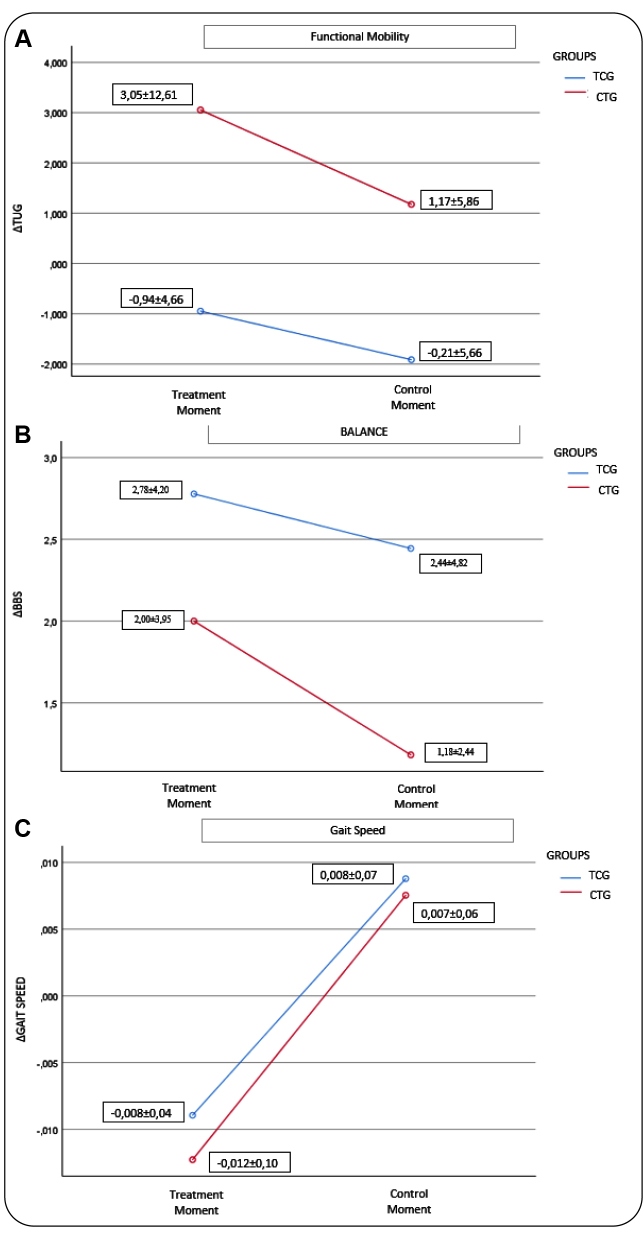
(**A)**. Comparison of mean values of functional mobility from ΔTUG* of human T-cell lymphotropic virus 1-associated myelopathy/tropical spastic paraparesis (HAM/TSP) between ET** and LT*** at treatment and control times (*p* = 0.290). **(B).** Comparison of mean values of ΔBBS**** balance of HAM/TSP between ET and LT at treatment and control times (*p* = 0.415). **(C).** Comparison of mean values of speed of HAM/TSP between ET and LT at treatment and control times (*p* = 0.296). *TUG= Timed up and Go; **ET= Early treatment;***LT= late treatment; ****BBS= Berg Balance Scale.

Our results indicate that the ET group demonstrated improvement in functional mobility and gait speed, but not balancing ability, which continued at the final evaluation point (i.e., after crossover); however, these differences were not significant. Conversely, we observed the worsening of functional mobility and gait speed in the LT group, despite interventions, over the 6-month period between initial and final evaluations (Figures 2A,B,C).

Comparisons between ET and LT interventions and control phases revealed a medium to large effect in functional mobility during the control phase. Patients in the LT group exhibited worse mean functional mobility scores than those in the ET group (Table 2).

**TABLE 2:** Estimation of the size effect in the early therapy and late therapy groups: intervention versus control phases.

Variable	Intervention phase	Control phase
Balance (∆BBS)	0.192 (−0.691-1.075)	0.341 (−0.547-1.228)
Functional mobility (∆TUG)	0.404 (−0.948-3.052)	0.553 (−0.345-1.45)
Gait speed change	0.039 (−0.842-0.92)	0.018 (−0.863-0.899)

*Cohen’s d.

## DISCUSSION

Our results indicate a medium to large effect with regard to functional mobility, as assessed using TUG in the ET group. Studies have demonstrated that rehabilitation involving VR video games gradually provides greater freedom of movement to patients, who reportedly feel safer owing to less fear of falling and can interact physically with ease with their surroundings^15^ ^,^ ^27^ ^-^ ^32^. In addition, studies have reported that rehabilitative treatment for sensorimotor impairment contributed to increased adaptive plasticity^33^ and that repetitive practice was positively associated with motor learning^34^ ^,^ ^13^.

Differences between performance and motor learning have already been described, and some factors, including repetition and motivation, favor knowledge retention and the development of motor memory^35^. Moreover, VR treatment protocols vary greatly among studies^30^. In rehabilitation programs involving patients with multiple sclerosis, the total number of sessions was 10-36^15^ ^,^ ^16^, scheduled 1-3 times a week^15^ ^,^ ^27^, each lasting from 20-60 min^27^ ^,^ ^32^. A literature review identified only one clinical trial involving HAM/TSP patients, employing 30-min VR rehabilitation sessions scheduled three times a week over 8 weeks^1^.

Studies indicate that impaired balance in individuals with myelopathy results from reduced muscle strength^35^ ^-^ ^39^ and spasticity in the lower limbs^2^ ^,^ ^35^ ^,^ ^36^. In addition, sensory changes and associated brain injuries can further compromise postural^38^ ^-^ ^43^and motor^44^ control, respectively. Notably, these sensory changes have been primarily associated with altered signal conduction through the ascending pathways in the thoracic spinal region, leading to reduced functional ability^43^. Memory consolidation and consequent motor learning are closely linked to motor and somatosensory information^45^.

The lower gait speed and inferior TUG performance seen in the LT group could be explained by the natural progression of myelopathy^46^, which leads to impairment in gait and mobility, eventually necessitating the use of walking aids^3^ ^,^ ^6^ ^,^ ^47^. Studies have linked functional mobility to several factors, including walking ability, weight transfer, balance, and muscle strength^21^, all of which are impaired in individuals with HAM/TSP^2^ ^,^ ^37^. Mobility presents as one of the greatest challenges for patients with HAM/TSP progression^36^, and over 70% of the affected women report reduced mobility and locomotion^3^. Gait speed was compromised in HAM/TSP patients, despite the use of walking aid devices^6^.

Our results contribute to the body of knowledge surrounding the therapeutic effects of VR video games in the rehabilitation of patients with HAM/TSP. This study has its limitations. The most notable limitation is the small sample size, which is unfortunately common in studies involving this type of myelopathy. Moreover, considering disease progression and the possibility of loss to follow-up, it would be advisable to employ a longer washout time. It would also be interesting to account for the possibility of fatigue in affected individuals when planning session duration times; investigating pain levels could lend further insight.

The size effect of VR videogaming on functional mobility was medium to large exclusively in the HAM/TSP group that underwent rehabilitation therapy earlier in our study.
